# Enhanced thermostability of a *Rhizopus chinensis* lipase by *in vivo* recombination in *Pichia pastoris*

**DOI:** 10.1186/1475-2859-11-102

**Published:** 2012-08-06

**Authors:** Xiao-Wei Yu, Rui Wang, Meng Zhang, Yan Xu, Rong Xiao

**Affiliations:** 1 State Key Laboratory of Food Science and Technology, Jiangnan University, 1800 Lihu Avenue, Wuxi, 214122, China; 2 Key Laboratory of Industrial Biotechnology, Ministry of Education, School of Biotechnology, Jiangnan University, 1800 Lihu Avenue, Wuxi, 214122, China; 3 School of Medicine and Pharmaceutics, Jiangnan University, Wuxi, 214122, China; 4 Center for Advanced Biotechnology and Medicine, Department of Molecular Biology and Biochemistry, Rutgers University, Piscataway, NJ, 08854, USA

**Keywords:** Lipase, Directed evolution, Thermostability, Mutant library construction, *Pichia pastoris*

## Abstract

**Background:**

Lipase from *Rhizopus chinensis* is a versatile biocatalyst for various bioconversions and has been expressed at high-level in *Pichia pastoris*. However, the use of *R. chinensis* lipase in industrial applications is restricted by its low thermostability. Directed evolution has been proven to be a powerful and efficient protein engineering tool for improvement of biocatalysts. The present work describes improvement of the thermostability of *R. chinensis* lipase by directed evolution using *P. pastoris* as the host.

**Results:**

An efficient, fast and highly simplified method was developed to create a mutant gene library in *P. pastoris* based on *in vivo* recombination, whose recombination efficiency could reach 2.3 × 10^5^ /μg DNA. The thermostability of r27RCL was improved significantly by two rounds of error-prone PCR and two rounds of DNA shuffling in *P. pastoris*. The S4-3 variant was found to be the most thermostable lipase, under the conditions tested. Compared with the parent, the optimum temperature of S4-3 was two degrees higher, *T*_m_ was 22 degrees higher and half-lives at 60°C and 65°C were 46- and 23- times longer. Moreover, the catalytic efficiency *k*_cat_/*K*_m_ of S4-3 was comparable to the parent. Stabilizing mutations probably increased thermostability by increasing the hydrophilicity and polarity of the protein surface and creating hydrophobic contacts inside the protein.

**Conclusions:**

*P. pastoris* was shown to be a valuable cell factory to improve thermostability of enzymes by directed evolution and it also could be used for improving other properties of enzymes. In this study, by using *P. pastoris* as a host to build mutant pool, we succeeded in obtaining a thermostable variant S4-3 without compromising enzyme activity and making it a highly promising candidate for future applications at high temperatures.

## Background

Lipases (triacylglycerol ester hydrolases EC 3.1.1.3) are enzymes that hydrolyze the ester bonds of water-insoluble substrates at the interface between substrate and water. They are well known because of their remarkable levels of activity and stability in non-aqueous environments, which makes them as attractive enzyme for use in industrial applications, such as in food, pharmaceutical, chemical and detergent industries [1-3].

*Rhizopus* lipases have been widely used in food industries [4,5]. Nevertheless, most *Rhizopus* lipases are mesozymes as they are produced by mesophilic organisms and exhibit unfavorable thermostability [6]. For example, lipase RAL from *R. arrhizus* showed poor thermostability when incubated at 30°C for 30 min [7]. Lipase ROL from *R. oryzae* showed highest activity at 35°C and about 35% of the lipase activity was lost after incubation at 45°C for 30 min [8]. Another lipase proROL derived from *R. oryzae* DSM853 exhibited better thermostability with a *T*_m_ value of 57.5°C [9]. In our previous study, a lipase gene *proRCL* from *R. chinensis* CCTCC M201021 was cloned and expressed at high-level in *Pichia pastoris* as r27RCL, whose activity was about 580 times higher than that of wild-type *R. chinensis* lipase [10]. This recombinant enzyme showed a high potential for industrial usage, as an additive for bread baking, synthesis of eicosapentaenoic acid (EPA), docosahexaenoic acid (DHA), sorbitan oleate, and ethyl esters [11-15]. However, the use of lipase r27RCL from *R. chinensis* is restricted by its low thermostability.

Directed evolution has been proven to be a powerful and efficient protein engineering tool for improvement of biocatalysts [16]. Several lipases from *Rhizopus* sp. were subjected to directed evolution in an effort to produce thermostable variants [7,17]. The half-life of RAL expressed in *P. pastoris* was prolonged by 12-fold at 50°C [7]. The *T*_m_ value of RNL from *R. niveus* expressed in *Saccharomyces cerevisiae* was improved from 47°C to 57°C [17].

The production of an active *Rhizopus* lipase has been performed in *Escherichia coli *[18], *S. cerevisiae *[19] and *P. pastoris *[20-22], among which the expression of *Rhizopus* lipase in *P. pastoris* was the best. The *P. pastoris* expression system offers several advantages, as a desirable host in directed evolution, for easy library screening and further industrial application, such as the ability to perform eukaryotic protein modifications, high levels of protein expression at the intra- or extracellular level and an *alcohol oxidase 1* (*AOX1*) gene promoter tightly regulated by methanol [23,24]. However, mutant library construction in *P. pastoris* usually requires time-consuming multistep procedures which include an intermediate bacterial host (*E. coli*) and restriction digestion procedures [25].

In this work, we developed a faster and simplified mutant library production method, based on which, the thermostability of the lipase r27RCL from *R. chinensis* CCTCC M201021 was improved by directed evolution.

## Results and discussion

### Library construction

**Figure 1:**
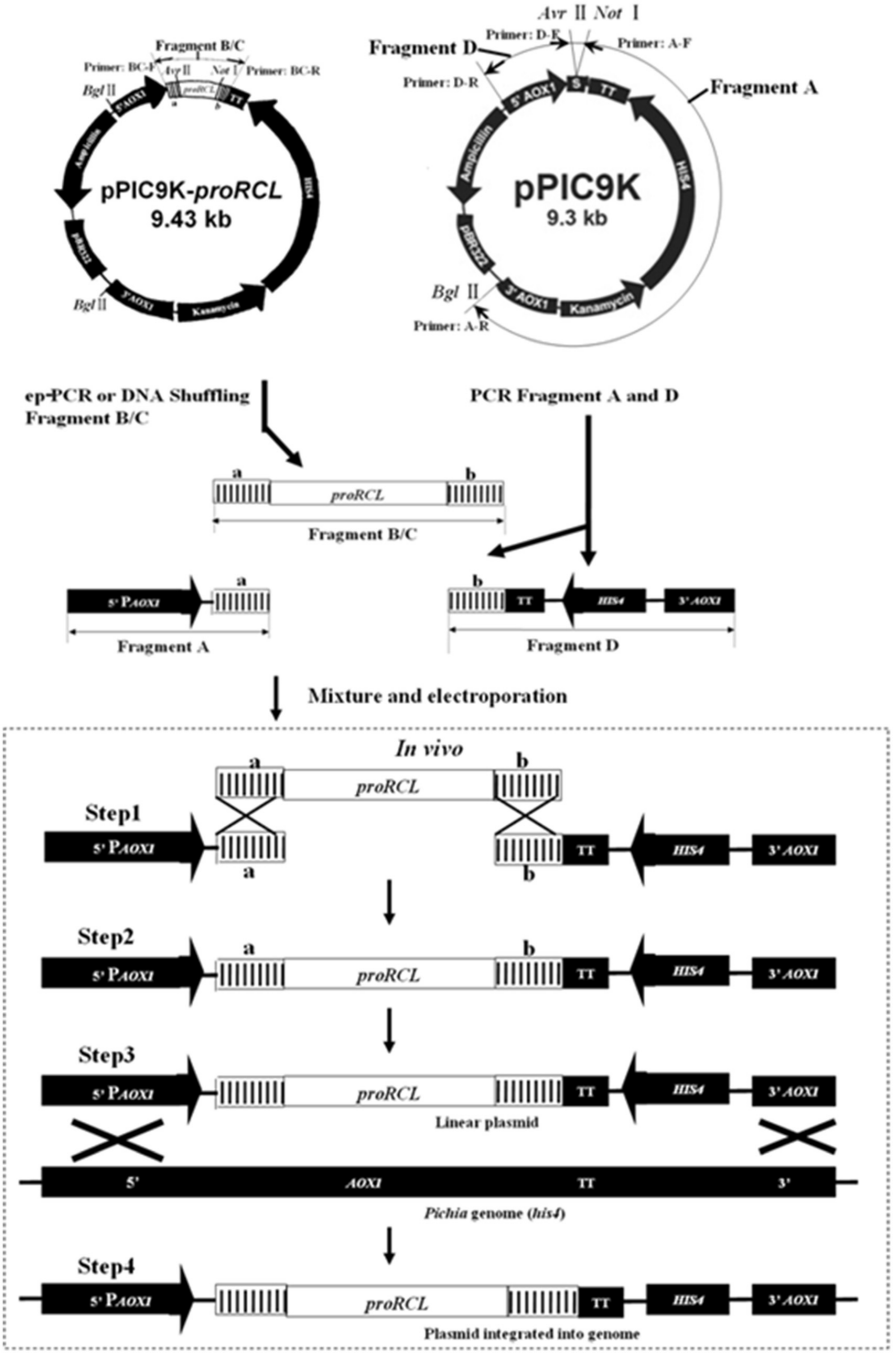
**The novel approach of construction of the lipase mutant library in *P. pastoris *based on the formation of a recombination cassette *in vivo *. **The hatched areas (**a**) and (**b**) were overlapped sequences between amplified fragments.

A novel approach for lipase mutant library construction in *P. pastoris* was established based on the formation of a recombination cassette *in vivo* (Figure 1). Three pairs of overlapping primers were used to generate the mutant gene (fragment B/C) and two different vector fragments (A and D). Primer BC-F had 66 bp overlapped pairs with the 5'-end of the gene sequence (“a” in Figure 1) of fragment D and primer BC-R had 60 bp overlapped pairs with the 3'-end of the gene sequence (“b” in Figure 1) of fragment A. Therefore, fragment B/C generated by primers BC-F and BC-R had 66 bp and 60 bp overlapped sequences with vector fragments D and A. The lipase gene fragment B (or C) and vector fragments A and D were mixed and transformed into *P. pastoris* competent cells by electroporation. *In vivo*, the fragments A, B/C and D self-reassembled to form a recombination cassette relying on the 60 bp and 66 bp homologous arms between them (Steps 1 and 2 in Figure 1). The self-reassembled expression cassette was integrated into the genome of *P. pastoris* by homologous recombination of the flanking *AOX1* gene sequences (Steps 3 and 4 in Figure 1).

Recombination efficiency is one of the most important factors in directed evolution. In order to improve the recombination efficiency, the effects of the fragment molar ratio and the length of the overlapping sequences for recombination were investigated. As shown in Table 1, the recombination efficiency was improved with the increased molar ratio of fragment B/C:A:D in the DNA mixture by electroporation. About 2.3 × 10^5^ /μg DNA recombinants were obtained when the molar ratio reached 10:1:1 and the values dropped to 1.8 × 10^5^ /μg DNA, 1.2 × 10^5^ /μg DNA or 1.0 × 10^5^ /μg DNA when the molar ratio was 5:1:1, 3:1:1 or 1:1:1. In order to obtain high recombination efficiency, the fragment molar ratio B/C:A:D of 10:1:1 was chosen. The recombination efficiency was low with shorter overlapping sequences (<55 bp) due to the difficulty in formation of a recombination cassette *in vivo*, while too long overlapping sequences (>70 bp) resulted in PCR technical difficulties with blurred and weak target bands when amplified with such long primers. Medium length of overlapping sequences (55-70 bp) generated satisfied recombination efficiency without PCR technical difficulties (Table 1). It is well known that homologous recombination is a common characteristic of yeast. [Baudin et al. 26] demonstrated that the amount of homology necessary to promote efficient recombination-mediated gene disruption in *S. cerevisiae* is in the order of 30–50 bp. [Oldenburg et al. 27] reported that 20 bp of homology is sufficient to generate the PCR-directed recombination in *S. cerevisiae*, at least under certain conditions (vector:insert 1:6). 

**Table 1:** The effects of the fragment molar ratio of B/C:A:D and the length of the overlapping sequences on recombination efficiency

**Method**		**Recombination efficiency **^**c**^**(× 10**^**5**^**, recombinants /μg DNA)**
Fragment B/C:A:D (molar ratio) ^a^	10:1:1	2.30
	5:1:1	1.80
	3:1:1	1.20
	1:1:1	1.05
Length of overlapping sequences (bp) ^b^	10	0.10
	25	0.80
	40	1.25
	55	1.75
	70	2.32
	85	2.35

^a^ The length of overlapping sequence was kept at 65 bp.

^b^ The fragment molar ratio of B/C:A:D was kept at 10:1:1.

^c^ Recombination efficiency is the number of recombinant cells (recombinants) generated by 1 μg of DNA fragments in a transformation procedure.

In this work, in order to make directed evolution practical in the host organism *P. pastoris* an efficient, fast and highly simplified method was developed to create a gene mutant library in *P. pastoris*. This method went a crucial step beyond previous approaches [25,28-30] because it completely discarded time-consuming multi-step procedures including construction of recombinant plasmid, transformation of an intermediate bacterial host (*E. coli*), plasmid isolation and linearization. In our approach, only three PCR amplification processes and an electroporation step were required. [Fernandez et al. 31] also reported a method to produce a mutant library in *P. pastoris*, which reassembled a recombination cassette *in vitro* and produced about 6000 colonies per 1 μg of linear DNA*.* The method developed in our research was more convenient and efficient since self-reassembly of the linear recombinant cassette occurred *in vivo*.

### Thermostable variants

**Figure 2:**
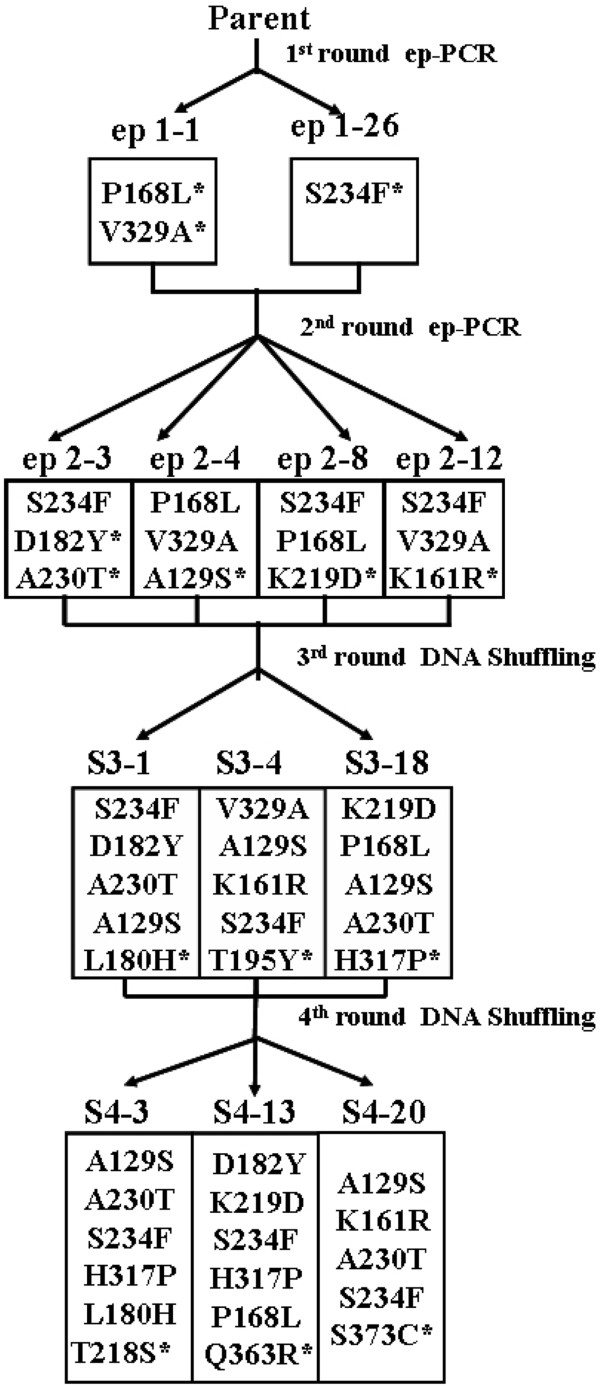
**Lineage of variants and amino acid substitutions by directed evolution. **Newly- introduced mutations in each generation are marked with asterisks.

The thermostability of r27RCL was improved by combining two rounds of error-prone PCR and two rounds of DNA shuffling (Figure 2). Error-prone PCR was used to introduce an average of 1 to 2 amino acid substitutions per *proRCL* gene and DNA shuffling could combine beneficial mutations as well as create new mutations. After creating the first-generation mutant library using error-prone PCR, approximately 5000 clones were screened for increased thermostability at 45°C. Two variants (ep1-1 and ep1-26) were identified and confirmed to have longer half-lives at 60°C (Table 2). ep1-1 (P168L, V329A) and ep1-26 (S234F) with a total of three substitutions were chosen as templates for generating the second-generation mutant library using error-prone PCR. Only 0.1% of the second generation mutant pool (5200 clones) screened at 50°C exhibited better performance than the first-generation parents. The identified variants ep2-3, ep2-4, ep2-8 and ep2-12 were selected for DNA shuffling. It was interesting to note that ep2-8 (S234F, P168L, K219D) and ep2-12 (S234F, V329A, K161R) both acquired mutations that were derived from ep1-1 and ep1-26 via *in vivo* DNA shuffling. From each generation of DNA shuffling, approximately 10000 clones were screened for increased thermostability. The third-generation evolution produced three variants (S3-1, S3-4, S3-18) whose thermostability slightly increased, whereas the last generation of evolution yielded three variants (S4-3, S4-13, S4-20) with significantly improved thermostability (Figure 2). S4-3 was the most thermostable variant. It contained five mutations (A129S, A230T, S234F, L180H, H317P) observed in previous generations plus one new mutation (T218S) generated by the fourth round of DNA shuffling.

**Table 2:** Thermostability of enzyme variants from each generation

**Generation**	**Variant**	***t***_**1/2**_**(min, at 60°C)**	**Fold Improvement**
0	parent	4.0	1.0
1	ep 1-1	9.8	2.4
	ep 1-26	8.7	2.2
2	ep 2-3	11.3	2.8
	ep 2-4	21.7	5.4
	ep 2-8	14.4	3.6
	ep 2-12	13.5	3.4
3	S 3-1	38.8	9.7
	S 3-4	58.9	13.9
	S 3-18	48.0	12.0
4	S 4-3	184.0	46.0
	S 4-13	168.3	42.1
	S 4-20	159.5	40.0

### Thermostabilities of the evolved lipases

Table 2 lists the *t*_1/2_ values of the parent and the evolved thermostable variants during four generations of directed evolution. At 60°C the *t*_1/2_ of the parent was 4 min. The variants from the first and the second generations had half-lives of thermal inactivation two- to five-fold longer than the parent. The half-lives of the three variants discovered from the third round of directed evolution (DNA shuffling) were 10 to 14 times longer than that of the parent at 60°C. The last round of mutagenesis resulted in three variants that had the longest half-lives of thermal inactivation, 40 to 46 times longer than the parent at 60°C, among which, S4-3 was the most thermostable enzyme. The enzymatic properties of the purified S4-3 were then studied and its thermo-mechanism was explored.

### Initial activity vs thermostability of variants

**Figure 3:**
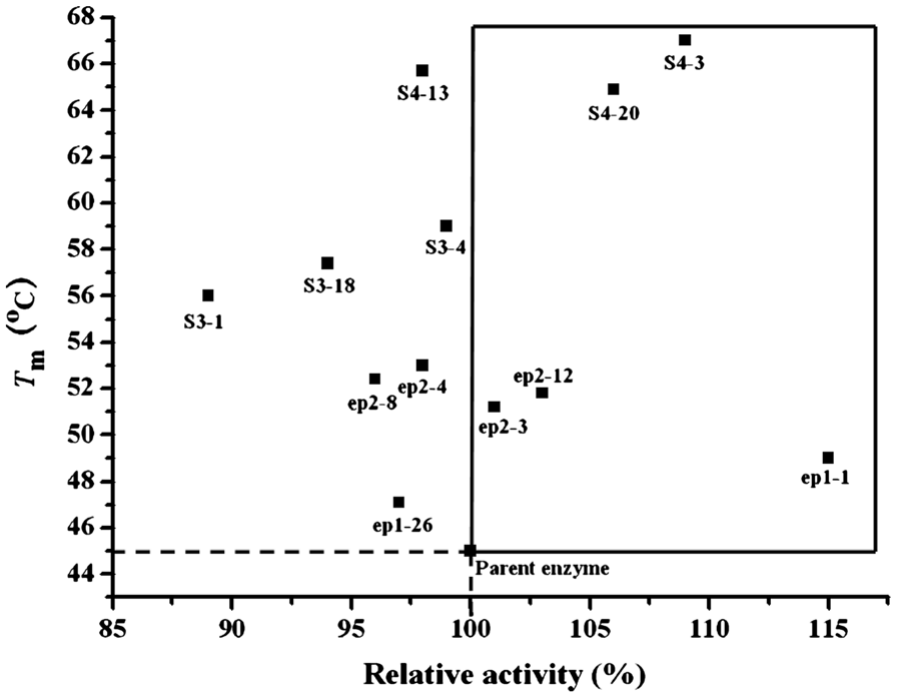
**Initial activity *vs T ***_**m **_**of the parent and variants. **The cross point of dashed lines and solid lines represent parent enzyme r27RCL. Variants exhibited higher activity and improved *T*_m _were squared with solid lines. The relative activity of the parent taking *p *NPP as substrate was set as 100%.

In order to determine the relationship between thermostability and enzyme activity, the initial activities of all 12 variants were measured. As shown in Figure 3, five variants exhibited improved *T*_m_ as well as higher activity. The activities of the other 7 variants were not affected much by increased thermostability, among which S3-1, with the lowest activity, still retained about 90% activity compared to the parent enzyme. These results provided strong evidence that improvement of thermostability and maintenance of enzyme activity could be simultaneously achieved, and improved thermostability did not occur at the expense of activity at low temperatures [32-34]. 

### Enzymatic properties of the thermostable variant S4-3

**Figure 4:**
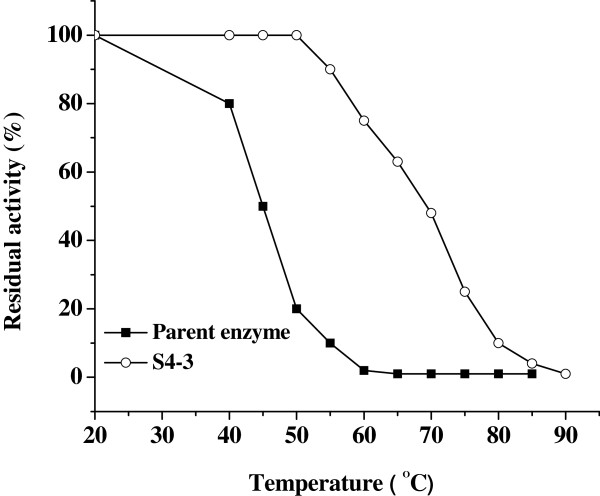
The effect of temperature on the stability of the parent and S4-3.

The most thermostable variant in the last generation, S4-3, was purified and its enzymatic properties were investigated. As shown in Figure 4, the residual activities of S4-3 and the parent were determined after incubation at different temperatures for 30 min. S4-3 displayed greatly- increased thermostability in the range of 20°C -60°C and retained 50% residual activity at 67°C, whereas the parent started to denature at 40°C and was completely inactivated at 60°C after 30 min heat-treatment.

**Figure 5:**
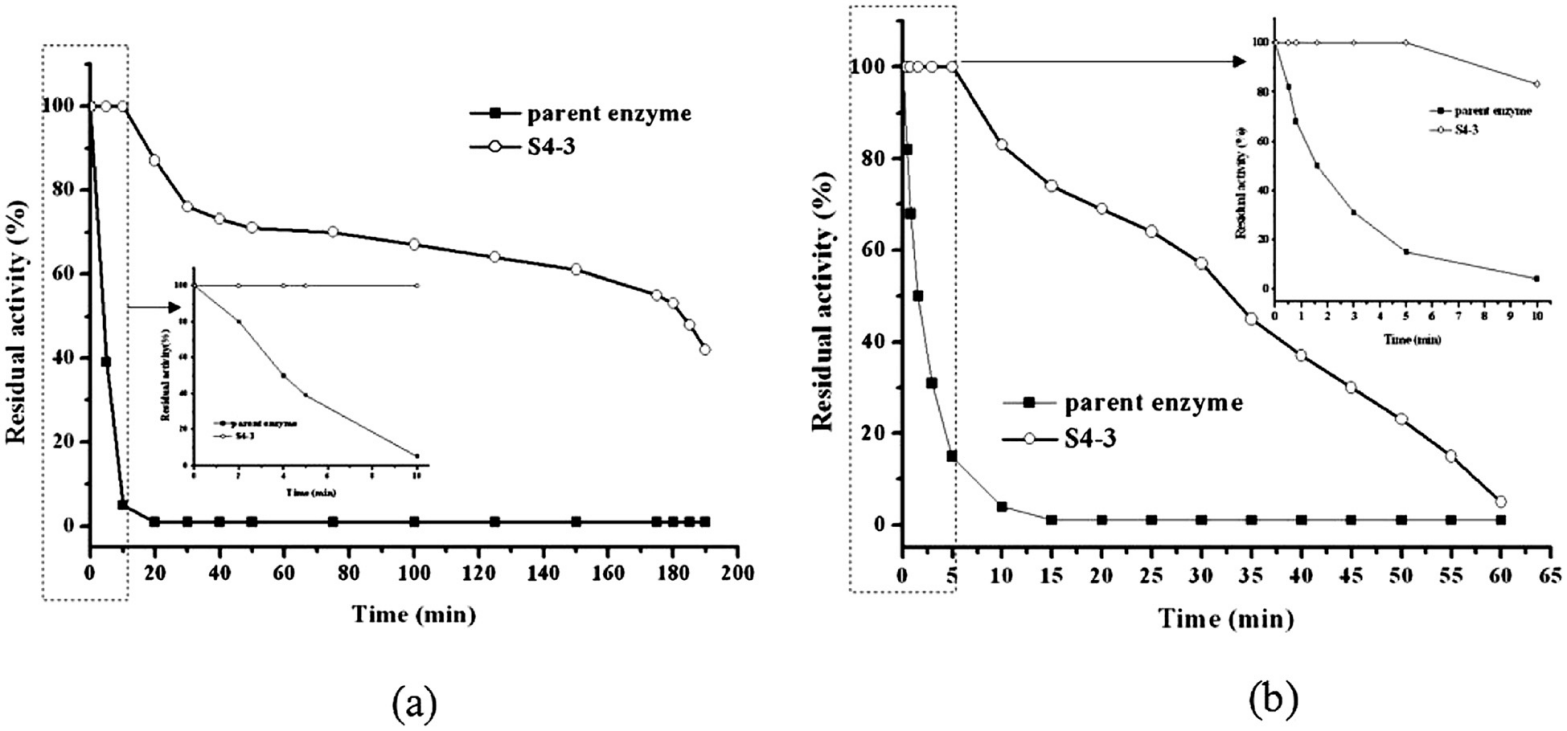
**Thermostability of the parent and S4-3 at 60° C (a) and 65°C (b).**

The thermal inactivation of S4-3 and the parent were characterized by incubation at 60°C and 65°C (Figure 5). The thermal inactivation curves of the parent at 60°C and 65°C were much steeper than those of S4-3, and the half-lives of S4-3 at 60°C and 65°C were 23- and 46-fold higher than those of the parent. In addition, the optimum temperature of S4-3 was also increased by 2°C compared with that of the parent (data not shown).

Kinetic parameters of the parent and S4-3 towards *p*NPP were calculated and listed in Table 3. S4-3 exhibited a 32% decrease in *K*_m_ whereas a 33% increase in *k*_cat_, resulted in almost identical catalytic efficiency *k*_cat_/*K*_m_ compared to the parent.

**Table 3:** Kinetic parameters of the parent and S4-3

**Enzyme**	***K***_**m**_**(mmol/min/mg)**	***k***_**cat**_**(s**^**-1**^**)**	***k***_**cat**_**/*K***_**m**_***(*M**^**-1**^** s**^**-1**^**)**
r27RCL	0.304	18.9	6.22 × 10^4^
S4-3	0.402	25.1	6.24 × 10^4^

### Thermo-mechanism of S4-3

**Figure 6:**
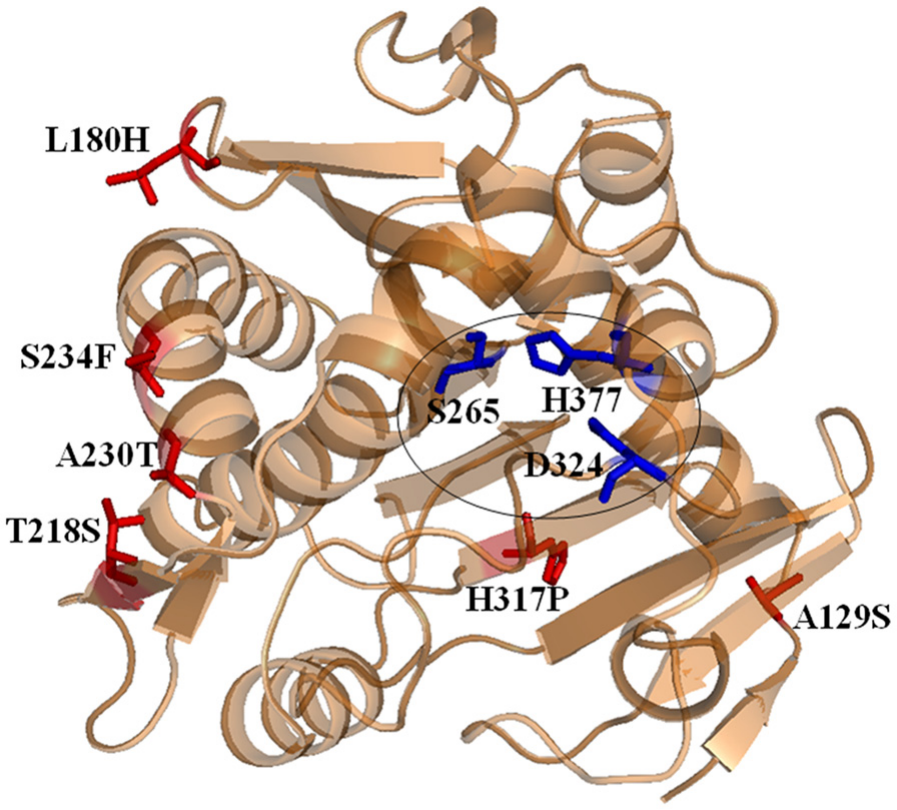
**Three-dimensional structure of r27RCL. **Locations of six amino acid substitutions (A129S, A230T, S234F, L180H, T218S, H317P) in S4-3 were shown in red sticks and the catalytic residues were shown in blue sticks (in the oval).

The three-dimensional homology model of r27RCL shows that the five mutations A129S, A230T, S234F, L180H, T218S were all present on the surface of the enzyme and far from the catalytic domain (S265-D324-H377) except for the mutation H317P (Figure 6). It is consistent with the notion that the initial steps in protein unfolding during the irreversible thermal denaturation primarily involve surface-located parts of the protein [35,36]. 

The contributions of mutations A230T and A129S toward thermostability could be interpreted based on the structure model which located them at the end of α-helix and in a loop on the protein surface. The substitution of A230 for T230 and A129 for S129 may have increased the hydrophilicity and polarity of the protein, which enhanced the thermostability of the enzyme [37]. Mutation L180H located in a loop on the protein surface introduced a positively-charged residue which may increase protein stability by creating a more favorable surface charge distribution [38,39], as found in engineered thermostable cold shock proteins [40]. The mutation S234F was located in an α-helix on the protein surface. It was surprising that replacement of an uncharged hydrophilic residue for a nonpolar hydrophobic residue (S234F) at the protein surface increased protein stability but this effect has been observed previously [40,41]. The H317P mutation is buried inside the protein in a β-strand. There are two assumptions that could be made to explain the improved thermostability. The first assumption was the addition of a hydrophobic residue. In the stabilized variant S4-3, hydrophilic histidine was replaced by hydrophobic proline, whose side-chain engaged in hydrophobic contacts with the side chain of Y293. This interaction was further improved by surrounding hydrophobic amino acids such as L137 and V341 and therefore enhanced protein stability. The alternative explanation was a reduction of the entropy of unfolding. The proline residue, with its pyrrolidine ring, had the lowest conformational entropy, thus the mutation histidine to proline should decrease the entropy of protein in the unfolded state and stabilize the protein [37].

It must be noted that the above-assumed thermo-mechanisms of S4-3 have been observed in many evolved thermostable enzymes and naturally-occuring thermophilic and hyperthermophilic proteins [35,42]. Determination of the crystal structure of S4-3 should further confirm its thermo-mechanism. But, the crystal structure of r27RCL has not been reported before. Even if it is available, it is still difficult to predict the specific thermostabilizing mutations based on its structure because there are so many structural features that stabilize proteins [37] and the contribution of each feature to the total free energy of thermostability is quite small.

## Conclusion

*P. pastoris* proved to be a valuable cell factory for the directed evolution of the lipase from *R. chinensis* for thermostability improvement, especially when combined with the novel method of library construction described here. In the present study, the thermostability of r27RCL was improved significantly by two rounds of error-prone PCR and two rounds of DNA shuffling in *P. pastoris*. The S4-3 variant is the most thermostable lipase from *Rhizopus* sp., under the conditions tested. Compared with the parent r27RCL, the optimum temperature of S4-3 was two degrees higher, *T*_m_ was 22 degrees higher and half-lives at 60°C and 65°C were 46- and 23- times longer, respectively. Moreover, the catalytic efficiency *k*_cat_/*K*_m_ of S4-3 was comparable to the parent. In summary, S4-3 maintained good catalytic properties and exhibited much greater tolerability and stability at high temperatures, which made this variant a promising candidate for industrial applications at high temperatures.

## Methods

### Strains, plasmid, reagents and cultures

Restriction endonucleases, *Taq* DNA polymerase were purchased from Takara. All chemicals were of analytical grade or higher quality and purchased from Sigma. *P. pastoris* GS115 (*His*^*-*^*Mut*^*+*^) and the pPIC9K expression vector are from Invitrogen BV. Recombinant plasmid pPIC9K-*proRCL* and strain GS115/ pPIC9K-*proRCL* were constructed by [Yu et al. 10]. The media used in this study include YPD (10g/L yeast extract, 20g/L peptone, 20g/L dextrose), MD (13.4g/L YNB, 0.016 μM biotin, 20g/L dextrose), BMGY (10g/L yeast extract, 20g/L peptone, 10g/L glycerol, pH 6.0 100 mM potassium phosphate, 13.4g/L YNB, 0.016 μM biotin), BMMY (10g/L yeast extract, 20g/L peptone, 10g/L methanol, pH 6.0 100 mM potassium phosphate, 13.4g/L YNB, 0.016μM biotin), BMMYA (10g/L yeast extract, 20g/L peptone, 5g/L methanol, pH 6.0 100 mM potassium phosphate, 13.4g/L YNB, 0.016 μM biotin, 6g/L agar).

### Production of proRCL variants by error-prone PCR

The first two rounds of random mutagenesis of *proRCL* gene were carried out by error-prone PCR as described elsewhere [43]. A 50 μL reaction mixture contained 5 μL PCR Gold buffer, 0.7 mM MnCl_2_, 3 mM MgCl_2_, 0.5 mM dATP/dGTP, 2.5 mM dCTP/dTTP, 40 pmol of each primer (BC-F and BC-R), 5 ng of plasmid pPIC9K-*proRCL*, and 2.5 U of *Taq* polymerase. PCR cycle conditions were: initial denaturation for 3 min at 94°C; followed by 30 cycles of 30 s at 94°C, 30 s of annealing at 55°C and 1 min of extension at 72°C; and a 10 min final extension at 72°C. The template for the second round of error-prone PCR used an equimolar mixture of positive variants from the first generation of random mutagenesis.

### Production of proRCL variants by DNA shuffling

The last two rounds of random mutagenesis of *proRCL* gene were carried out by DNA shuffling based on the previously methodology reported by [Zhao and Arnold 44]. The DNA template was an equimolar mixture of positive variants from the former round. The template was digested with 0.1 U DNase I in 200 μL of 50 mM Tris-HCl pH 7.4, 1 mM MgCl_2_ for 10 min at 37°C. Fragments of 50-100 bp were purified by gel extraction (QIAGEN). Then purified fragments were resuspended in PCR mixture (without primers) containing 5 μL PCR Gold buffer, 200 μM of each dNTP and 1.25 U of *Taq* polymerase. PCR cycle conditions were: initial denaturation for 3 min at 94°C; followed by 10 cycles of 30 s at 94°C, 30 s of annealing at 45°C and 1 min of extension at 72°C; 10 cycles with annealing temperatures of 48°C and 51°C, respectively; and a 10 min final extension at 72°C. Then, 1-3 μL of primerless PCR product was added to a 50 μL PCR mixture with 2 μM primers (BC-F/BC-R). Other PCR conditions were similar to those described above, and a single product comprising full-length *proRCL* was obtained.

### Production of his4 3'-end containing fragment (vector fragment A and D)

Expression vector fragments were prepared by PCR amplification. The plasmid pPIC9K-*proRCL* was first amplified using flanking primers A-F/A-R or D-F/D-R to produce vector fragment A or D (Figure 1). The PCR was carried out in a final volume of 50 μL containing a mixture of 50 ng plasmid pPIC9K-*proRCL*, 1 μM of each primers, 5 μL PrimeSTAR DNA polymerase buffer, 100 μM of each dNTP and 1.25 U of PrimeSTAR DNA polymerase. PCR cycle conditions were: initial denaturation for 3 min at 94°C; followed by 30 cycles of 30 s at 94°C, 30 s of annealing at 50°C and 2.5 min or 7.5 min extension at 72°C for fragment D or A, respectively; and a 10 min final extension at 72°C.

### Library construction

*P. pastoris* was transformed with 2 μg of fragments A, D and B/C in a 10:1:1 molar ratio by electroporation according to the protocol reported by [Wu and Letchworth 45]. Briefly, the competent cells, pretreated with 0.1 M lithium acetate and 10 mM dithiothreitol, were transformed with 2 μg DNA fragments by electroporation with instrument settings of 1.5 kV, 25 μF and 186 Ω. Transformed yeast colonies appeared in 4-6 days at 30°C on MD plates. The variants were confirmed by DNA sequencing (Sangon Biotech Company).

### Overlapping sequence length

In order to optimise the recombination efficiency, the length of the overlapping sequences between vector and gene fragments was optimized. It was noteworthy that the lipase gene fragment was be produced by normal PCR (called L) instead of ep-PCR. We generated fragment L using 10 bp, 25 bp, 40 bp, 60 bp, 70 bp and 85 bp of overlapping primers listed in Table 4. These fragments were mixed with fragment A and D in a 10:1:1 molar ratio and electroporated into *P. pastoris*.

**Table 4:** Oligonucleotides utilized in this study

**Primer**	**Oligonucleotide sequence**
BC-F	GCTGCTAAAGAAGAAGGGGTATCTCTCGAGAAAAGAGAGGCTGAAGCTTACGTAGAATTCCCTAGG
BC-R	GTAAGTGCCCAACTTGAACTGAGGAACAGTCATGTCTAAGGCGAATTAATTCGCGGCCGC
A-F	GGCCGCGAATTAATTCGCCTTAGACATG
A-R	AGATCTTGATATAAATTTCACGTTTAAAATC
D-F	TCGACAATTGGTTTGACTAATTCCATAATCTG
D-R	ACCTTTCGTCTTTGGATGTTAGTCT
BC-F10	ATTCCCTAGG
BC-R10	TCGCGGCCGC
BC-F25	TGAAGCTTACGTAGAATTCCCTAGG
BC-R25	CTAAGGCGAATTAATTCGCGGCCGC
BC-F40	CGAGAAAAGAGAGGCTGAAGCTTACGTAGAATTCCCTAGG
BC-R40	GAGGAACAGTCATGTCTAAGGCGAATTAATTCGCGGCCGC
BC-F55	GAAGGGGTATCTCTCGAGAAAAGAGAGGCTGAAGCTTACGTAGAATTCCCTAGG
BC-R55	GCCCAACTTGAACTGAGGAACAGTCATGTCTAAGGCGAATTAATTCGCGGCCGC
BC-F70	CATTGCTGCTAAAGAAGAAGGGGTATCTCTCGAGAAAAGAGAGGCTGAAGCTTACGTAGAATTCCCTAGG
BC-R70	CGGTCTTCTCGTAAGTGCCCAACTTGAACTGAGGAACAGTCATGTCTAAGGCGAATTAATTCGCGGCCGC
BC-F85	TACTACTATTGCCAGCATTGCTGCTAAAGAAGAAGGGGTATCTCTCGAGAAAAGAGAGGCTGAAGCTTACGTAGAATTCCCTAGG
BC-R85	AGAATCTAGCAAGACCGGTCTTCTCGTAAGTGCCCAACTTGAACTGAGGAACAGTCATGTCTAAGGCGAATTAATTCGCGGCCGC
BC-F65	CTGCTAAAGAAGAAGGGGTATCTCTCGAGAAAAGAGAGGCTGAAGCTTACGTAGAATTCCCTAGG
BC-R65	TTCTCGTAAGTGCCCAACTTGAACTGAGGAACAGTCATGTCTAAGGCGAATTAATTCGCGGCCGC

The number in the name of the primers indicated the length of the overlapping sequences.

### Pre-screening

Colonies from libraries of each generation were replicated with sterile toothpicks from MD plates onto YPD plates (for the storage of variants) and BMMYA plates (for gene expression and screening), simultaneously. The parent strain was used as a positive control. YPD and BMMYA plates were then incubated at 28-30°C for 2 d and 5 d, respectively. Then, BMMYA plates from each ep-PCR and DNA shuffling generation were subjected to heat-treatment at 45°C, 50°C, 55°C and 65°C, respectively, for 60 min. Screening was performed with 15 mL of top agar (5g/L) containing 160 μL of Fast blue RR (89g/L in dimethyl sulfoxide) and 80 μL α-naphthyl acetate (40g/L in dimethyl formamide) [46]. Positive colonies exhibited dark brown coloration within two minutes after pouring the top agar and were further screened using the 96 MTPs screening method.

### Screening for evolved variants

All positive candidates screened from top-agar staining were picked and cultivated in deep-well microtiter plates containing 900 μL of BMGY medium, at 28°C, 300 rpm for 16 h to reach an *OD*_600_ of 1. Then, cultures were harvested by centrifugation at 3000 g at 4°C for 10 min and the cells were suspended in 300 μL of BMMY medium for gene expression. The cultures were kept at 28°C, 300 rpm for 84 h with supply of 100 μL fresh BMMY medium and 10g/L methanol every 24 h.

Each culture was centrifuged at 5000g at 4°C for 10 min. The supernatant was then transferred into 96-well microtiter plates for heat-treatment at 60°C or 65°C for 60 min. Initial and residual lipase activities were measured. All the assays were done in triplicate and significant differences (p *<* 0.05) were measured. Only variants that showed both higher ratio of residual activity and similar initial activity compared to the parent enzyme were selected.

### Enzyme purification

A single colony from each variant was cultivated in 100 mL of BMGY medium shaken at 28°C and 250 rpm in 500 mL glass flasks. When cultures reached an *OD*_600_ of 1, the cells were centrifuged and resuspended in 25 mL of BMMY medium to obtain an *OD*_600_ of 4 and shaken at 28°C and 250 rpm in 250 mL glass flasks for 84 h. The cultures were supplemented with methanol (5g/L) to induce the expression of lipase every 12 h. The culture was then centrifuged and the supernatant was collected for protein purification.

Cell free medium was concentrated and interchanged with 10 mM Tris-HCl buffer (pH 7.5) by ultra filtration through a 10-kDa membrane (Millipore). The concentrated solution was loaded onto a SP-Sepharose column (AKTA, 1 cm × 20 cm) equilibrated with 20 mM Tris-HCl buffer (pH 7.5) and eluted with 0-0.5 M NaCl in the same buffer. Fractions containing lipase activity were pooled, concentrated and loaded on a Phenyl-sepharose 6 FF column (AKTA, 1.6 cm × 20 cm) equilibrated in 50 mM Tris-HCl buffer (pH 7.5) containing 1.6 M ammonium sulfate. Lipase was then eluted in an ammonium sulfate concentration gradient decreasing from 1.6 to 0 M in 50 mM Tris-HCl buffer (pH 7.5) and 4 mL fractions were collected at a flow rate of 0.8 mL/min.

### Lipase properties

Lipase activity was measured on emulsified *p-*nitrophenyl palmitate (*p*NPP) according to [Kordel et al. 47]. Unless stated otherwise, the typical enzymatic reaction was carried out at 40°C, pH 8.5. One enzyme unit was defined as the amount of enzyme releasing 1 μmol of *p*-nitrophenol per minute under the assay conditions. Optimal temperature (*T*_opt_) was determined by measuring the enzyme activity at pH 8.5 under various temperatures (20-60°C). *T*_m_ values were determined as described previously [43]. 10 U purified enzyme solutions were incubated for 30 min in the temperature range of 20-80°C, cooled in the ice bath for 20 min and equilibrated at room temperature for 5 min. Then, residual activity was determined at 40°C and expressed as a percentage of the initial activity. The temperature at which 50% of lipase activity lost was the *T*_m_ value. Purified enzymes were incubated at specific temperatures (60°C and 65°C) for half-life (*t*_1/2_) determination. 10 U purified enzyme solutions were taken at various time intervals, cooled in the ice bath for 20 min and equilibrated at room temperature for 5 min. Then, residual activity was determined and the incubation time at which 50% of lipase activity lost was the *t*_1/2_ value. Inactivation was followed until > 80% of the activity was lost. The Michaelis-Menten kinetic parameters *k*_cat_ and *K*_m_ were calculated using *p*NPP as substrate. Lineweaver-Burk plots were used to determine *k*_cat_ and *K*_m_ parameters, assuming that the reactions followed a simple Michaelis-Menten kinetics.

### Thermo-mechanism analysis

A three-dimensional model of *R. chinensis* lipase was built by SWISS-MODEL protein automated modelling program [48] on the basis of crystal structure of lLGY (crystal structure of lipase II from *Rhizopus niveus* solved with a resolution of 2.20 Å) [49], which showed the highest homology of 80.38% to *R. chinensis* lipase.

## Abbreviations

96 MTPs: 96 micro test plates; *p *NPP: *p *-nitrophenyl palmitate.

## Competing interests

The authors declare that they have no competing interests.

## Authors’ contributions

Yu made substantial contributions to design the experiments and draft the manuscript. Wang carried out this research work, interpreted the data and drafted the manuscript. Zhang carried out the purification experiment. Xu and Xiao revised the manuscript. All authors read and approved the final manuscript.
